# TAS-303 effects on urethral sphincter function in women with stress urinary incontinence: phase I study

**DOI:** 10.1007/s00192-020-04470-7

**Published:** 2020-10-10

**Authors:** Makoto Yono, Shin Irie, Momokazu Gotoh

**Affiliations:** 1 Department of Urology, Nishi Kumamoto Hospital, Souseikai, Kumamoto, Japan; 2 1012 Koga, Tomiai-machi, Minami-ku, Kumamoto, 861-4157 Japan; 3 Department of Clinical Pharmacology, Nishi Kumamoto Hospital, Souseikai, Kumamoto, Japan; 4 Present Address: Souseikai, Fukuoka, Japan; 5 Department of Urology, Nagoya University Graduate School of Medicine, Aichi, Japan; 6 Present Address: Chukyo Hospital, Aichi, Japan

**Keywords:** Japan, Maximum urethral closure pressure, Randomized controlled study, Stress urinary incontinence, TAS-303, Urethral pressure profile parameters

## Abstract

**Introduction and hypothesis:**

TAS-303, which selectively inhibits noradrenaline reuptake, was developed for treating stress urinary incontinence (SUI). The proximal urethra mainly comprises smooth muscle fibers in which α1 adrenergic receptors are abundant. This study was conducted to evaluate the effect of TAS-303 on urethral function and its safety profile in female patients with SUI.

**Methods:**

In total, 16 women (age, 20–64 years) with SUI and > 5.0 g of leakage in the 1-h pad test at screening were randomized and administered the assigned treatment in a double-blind manner. The primary end point was change in the maximal urethral closure pressure (MUCP) at 6 h post-dose. The secondary end point was change in the urethral closure pressure of the entire urethra and each urethral region (proximal, middle, and distal) at 6 h post-dose. The results were analyzed using a t-test.

**Results:**

The mean change ± standard deviation in MUCP at 6 h post-dose was 3.473 ± 12.154 cmH_2_O for TAS-303 and 2.615 ± 9.794 cmH_2_O for placebo (between-group difference: 0.858 cmH_2_O, *P* = 0.8047). The mean changes ± standard deviation in urethral closure pressure of the proximal urethra at 6 h after the administration of TAS-303 18 mg and placebo were 3.863 ± 10.941 and 1.634 ± 12.093, respectively (between-group difference: 2.229 cmH_2_O, *P* = 0.5976).

**Conclusions:**

No significant difference in MUCP and urethral closure pressure was found between TAS-303 and placebo. However, the change in the proximal urethral closure pressure with TAS-303 was larger than that with placebo. This suggests that TAS-303 has pharmacological effects on urethral sphincteric function.

**Electronic supplementary material:**

The online version of this article (10.1007/s00192-020-04470-7) contains supplementary material, which is available to authorized users.

## Introduction

Stress urinary incontinence (SUI), defined as a “complaint of involuntary loss of urine on effort or physical exertion or on sneezing or coughing” by the International Continence Society [1], is a common health problem among women and has a significant negative impact on quality of life [2–4].

According to the clinical guidelines on urinary incontinence in the European Union [5, 6] and female lower urinary tract symptoms in Japan [7], for all female patients with SUI, initial management includes pharmacotherapy as well as lifestyle interventions and pelvic floor muscle training. Indeed, duloxetine (approved in the European Union) and clenbuterol (approved in Japan) are used as a treatment for women with SUI in the European Union and Japan, respectively. Female patients who have failed initial management and are bothered by their symptoms and an impaired QoL are likely to request further treatment, e.g., surgery. It is thus conceivable that if initial management has been exhausted, surgery for SUI may be indicated. However, owing to the difficulty in performing long-term pelvic floor muscle exercises, as well as concerns regarding the invasiveness and safety of surgery, the clinical need for a new SUI treatment remains unmet [8, 9].

TAS-303, developed by Taiho Pharmaceutical for treating SUI, selectively inhibits noradrenaline reuptake without centrally mediated antidepressant activity. In animal studies, TAS-303 increased the urethral basal pressure in normal rats and increased the leak point pressure in a rat vaginal distension model, suggesting its potential efficacy for treating SUI [10].

Considering the mechanism of action (increasing the urethral pressure) of TAS-303, the maximum urethral closure pressure (MUCP) was presumed to be an appropriate urodynamic parameter and was determined to be the primary study end point according to Section 6.2.3 (“Stress incontinence”) of the European Medicine Agency clinical investigation guideline [11]. MUCP can be used in proof-of-mechanism studies of new drugs developed to treat SUI [12]. The proximal urethra mainly consists of smooth muscle fibers in which α1 receptors are abundant [13, 14]. Therefore, we considered the proximal urethra appropriate for evaluating the mechanism of action of TAS-303.

The primary aim of this phase I study was to evaluate the effects of a single oral dose (18 mg) of TAS-303 on MUCP in women with SUI. The secondary objectives were to assess the effects of TAS-303 on the MUCP of different urethral regions and also to investigate the pharmacokinetics (PK) and the safety of TAS-303.

## Materials and methods

### Study design and treatment

This was a single-center, randomized, double-blind, placebo-controlled, two-period cross-over, single-dose, phase I study conducted in the period from October to December 2015 at Nishi Kumamoto Hospital in Japan. The study design is shown in Supplementary Fig. 1. Sixteen patients with predominant SUI were randomized to either group A (TAS-303 18 mg followed by placebo) or group B (placebo followed by TAS-303 18 mg) at a 1:1 ratio (eight patients per group). The study comprised two periods in which TAS-303 18 mg or placebo was administered as a single oral dose in a double-blind manner. In period 1, patients deemed eligible for the study at screening were hospitalized the day before study drug administration (day −1), administered a single oral dose of the study drug on day 1, and discharged from the study site with no identified safety concerns on day 4. Measurement of urethral closure pressure was conducted before administration and 6 h post-dose. Subsequently, period 2 was started and followed the same procedures as period 1 (admission to the study site on day 14 followed by a single administration of the study drug on day 15 and discharge on day 18). Between 14 and 21 days post-dose in period 2, the patients returned to the study site for follow-up evaluation. The patients were not permitted to lie down for 4 h post-dose unless this was needed for clinical assessments.

To prevent infections, levofloxacin was administered as a single oral dose of 500 mg after each measurement of urethral pressure. The prohibited concomitant medications and therapies and the details of randomization and blinding are described in Supplementary Methods.

This trial was conducted in accordance with the Good Clinical Practice Guidelines and the Declaration of Helsinki. The protocol and informed consent form were approved by the Institutional Review Board at Hakata Clinic (Souseikai Global Clinical Research Center) in Japan. Written informed consent was obtained from all study participants. This study was registered at ClinicalTrials.gov (NCT02562807).

### Patients

Women aged 20 to < 65 years with predominant SUI, body mass index (BMI) of 18.0 to <3 0.0 kg/m^2^, and leakage of > 5.0 g in the 1-h pad test at screening were included in this study. Patients with predominant urge urinary incontinence, a history of surgical therapy for SUI, and/or presence of pelvic organ prolapse were excluded.

### Efficacy endpoints

The primary end point was the change in MUCP at 6 h post-dose. The secondary end points were urethral pressure profile (UPP) parameters, including changes in MUCP and mean urethral closure pressure at rest for the entire urethra and for the proximal, middle, and distal thirds of the functional urethral length at 6 h post-dose. The measurement time of MUCP was 6 h post-dose because C_max_ was 6 h when 18 mg was administered in a single-dose phase I study in healthy adult males (unpublished data).

The functional urethral length was automatically divided into three sections using Duet software (Mediwatch, Rugby, UK). The proximal, middle, and distal thirds of the urethra were defined as 30%, 40%, and 30% of the total length of the functional urethra, respectively.

The mean and maximum values for each UPP parameter were calculated for the entire urethra and for individual regions. The UPP parameter was measured three times. The mean and maximum values calculated from three successive profiles were averaged within each patient.

### Safety end points

Adverse events (AEs) and adverse drug reactions, physical findings, blood pressure, pulse rate, body temperature, 12-lead electrocardiography findings, and laboratory test results were evaluated to determine the safety of TAS-303. AE data were collected and coded using MedDRA® (version 18.1) terms. The severity of AEs was evaluated and classified using Common Terminology Criteria for Adverse Events version 4.0, Japan Clinical Oncology Group edition version 4.03.

### PK analysis

The plasma concentrations of TAS-303 were measured at Shin Nippon Biomedical Laboratories, Ltd., using validated liquid chromatography/tandem mass spectrometry. The following PK parameters of TAS-303 were evaluated using Phoenix® WinNonlin® (version 6.4; Certara, LP, Princeton, NJ, USA): maximum plasma concentration (C_max_), time to maximum plasma concentration (t_max_), area under the plasma concentration-time curve from time zero to the final time of detection (AUC_0-last_), area under the plasma concentration versus time curve from time zero to infinity (AUC_0-inf_), and elimination half-time (t_1/2_).

### Urethral pressure measurement

Urethral pressure was measured in accordance with the Report from the Standardization Subcommittee of the International Continence Society [15]. The UPP parameters at rest were measured before and 6 h after administering TAS-303 18 mg and placebo. Three successive profiles were measured with the patient resting and in the absence of bladder stimulation. Additional details are provided in Supplementary Methods.

### Statistical analysis

The target sample size of 16 patients was not statistically determined considering the invasiveness of this urethral pressure measurement method. Data are shown as mean ± standard deviation. Summary statistics for the changes in MUCP in the entire urethra and in each urethral region and two-sided 95% confidence intervals (CIs) were calculated with respect to each group. Differences in the changes in MUCP between groups were analyzed using a t-test. The safety analysis set comprised all randomized patients who received one dose of the study drug. The primary and secondary efficacy end points were assessed in the full analysis set, comprising all randomized patients who received at least one dose of the study drug, met all the inclusion criteria and none of the exclusion criteria, and had primary end point data at periods 1 and 2. Data were analyzed using SAS software, version 9.2 (SAS Institute, Cary, NC, USA).

## Results

**Fig. 1:**
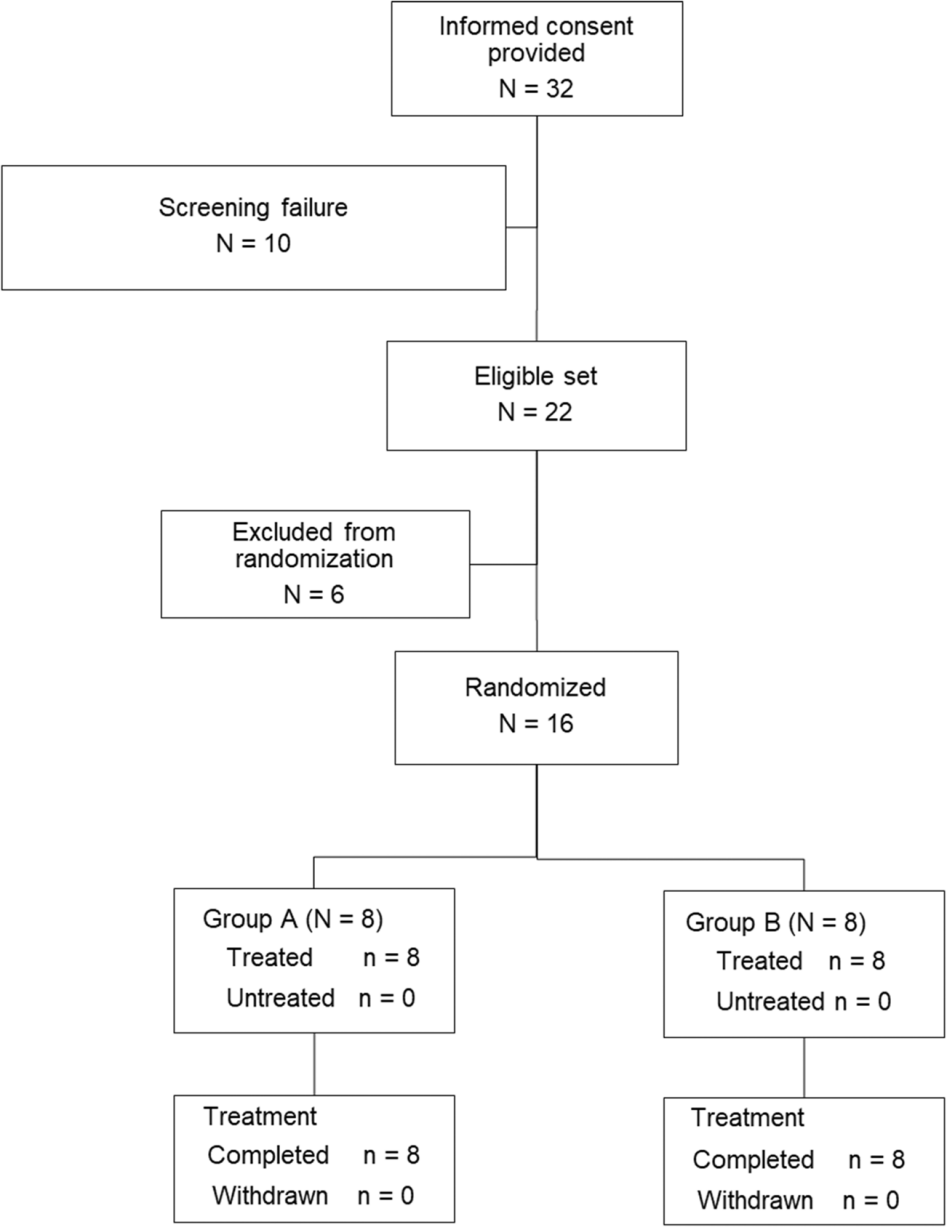
Disposition of patients with SUI

The flow of patient disposition is shown in Fig. 1. Thirty-two patients provided informed consent; 22 patients were eligible at screening; 16 women were randomized. All randomized patients received the study drug: eight were assigned to group A (TAS-303 18 mg followed by placebo) and eight were assigned to group B (placebo followed by TAS-303 18 mg). All patients completed the study.

The median age was 53.0 (range 23–62) years, and the median BMI was 20.69 (range: 18.5–29.7) kg/m^2^. Ten patients had SUI, and six had mixed urinary incontinence (Supplementary Table 1).

### Efficacy

#### Urethral pressure at rest

The mean change ± standard deviation (95% CI) in MUCP at 6 h post-dose (primary end point) was 3.473 ± 12.154 (−3.003 to 9.949) cmH_2_O for TAS-303 18 mg and 2.615 ± 9.794 (−2.604 to 7.834) cmH_2_O for placebo, with a between-group difference of 0.858 cmH_2_O (95% CI, −6.444 to 8.160; *P* = 0.8047).

#### Effect of TAS-303 on different urethral regions

**Fig. 2:**
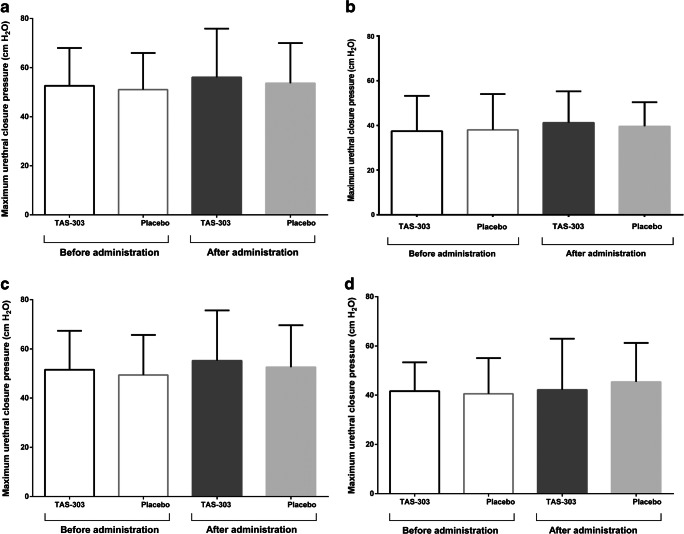
Maximum urethral closure pressure at rest for the entire urethra (**a**) and for the proximal (**b**), middle (**c**), and distal urethral region (**d**) before and after administration of TAS-303 18 mg (*n* = 16) or placebo (*n* = 16)

**Fig. 3:**
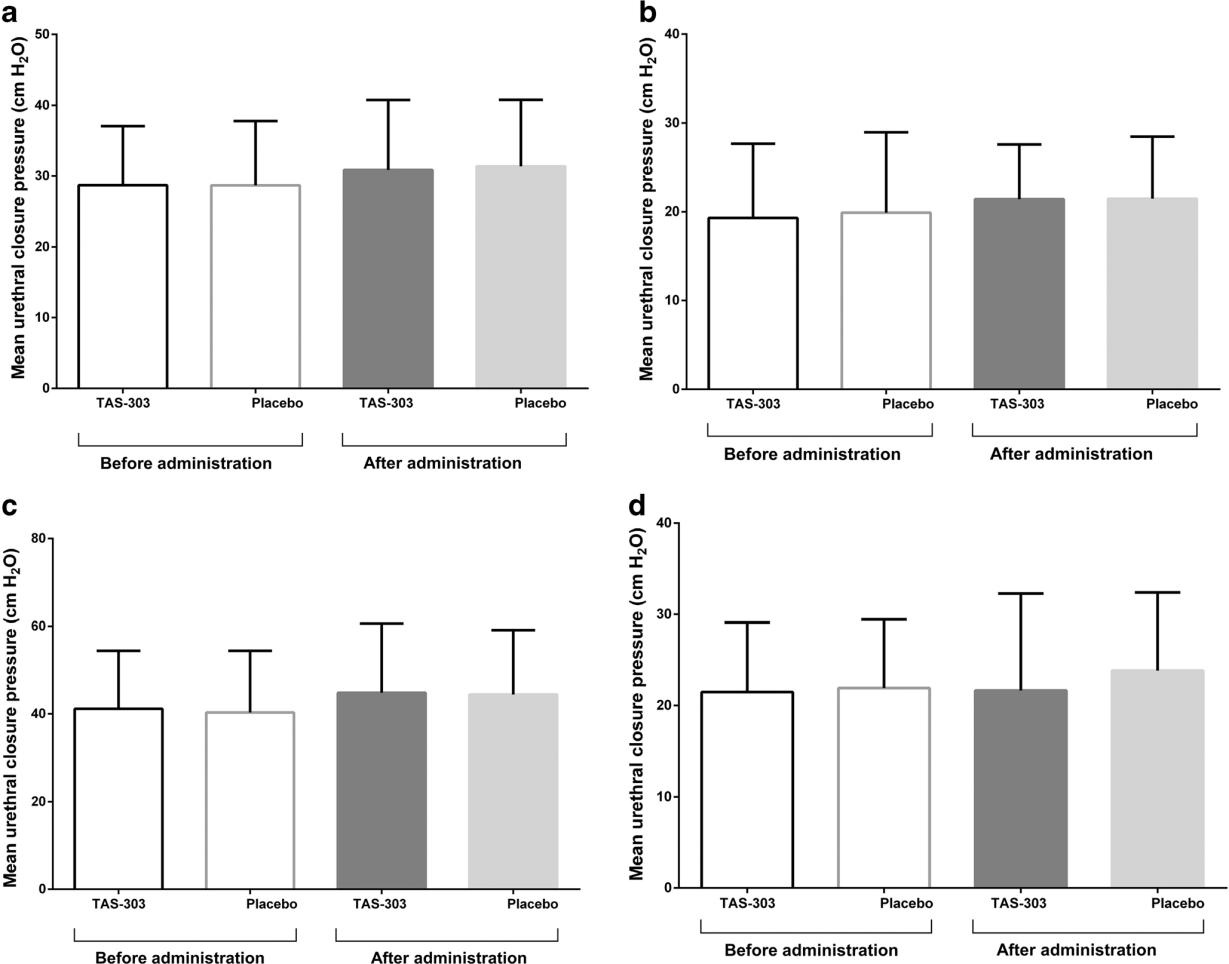
Mean urethral closure pressure at rest for the entire urethra (**a**) and for the proximal (**b**), middle (**c**), and distal urethral region (**d**) before and after administration of TAS-303 18 mg (*n* = 16) or placebo (*n* = 16)

When the MUCP (Fig. 2) and mean urethral closure pressure (Fig. 3) at rest were separately analyzed for the proximal, middle, and distal thirds of the functional urethral length, no significant difference was found between TAS-303 and placebo in any region. However, among the three regions, the proximal part of the urethra, where α1 receptors are concentrated, showed the largest change, and the mean change in MUCP from before to after administration was larger with TAS-303 than with placebo (TAS-303: 3.863 ± 10.941 [−1.967 to 9.693], placebo: 1.634 ± 12.093 [−4.809 to 8.078]).

### Safety

No deaths or other serious AEs were reported, and no patients were withdrawn from the study because of AEs. The only AE observed in this study was a ligament sprain in one patient who received TAS-303 18 mg, and this AE was considered to be unrelated to the study drug. No adverse drug reactions were reported for TAS-303 18 mg. No clinically significant abnormal changes were observed in laboratory data (hematology, blood chemistry, and urinalysis). Similarly, no clinically significant abnormal changes were observed in heart rate, pulse rate, and blood pressure.

### PK analysis

**Fig. 4:**
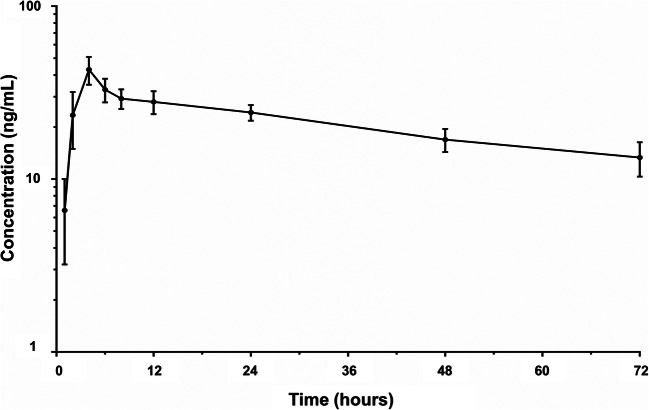
Mean plasma concentration-time profile of TAS-303 in women with stress urinary incontinence (semi-logarithmic scale) after administration of TAS-303 18 mg. *N* = 16, arithmetic mean ± standard deviation

The mean plasma concentration-time profile of TAS-303 is shown in Fig. 4. The PK parameters of TAS-303 administered as a single oral dose of 18 mg under fasting conditions in patients with SUI are shown in Table 1.

**Table 1:** PK parameters of TAS-303 in patients with SUI

	C_max_ (ng/ml)	t_max_ (h)	AUC_0–inf_ (ng•h/ml)	t_1/2_ (h)
Geometric mean	42.341	4.0 (4.0–8.0)*	2677	57.76
Geometric coefficient of variation (%)	20.6	NA	31.4	41.6
N	16	16	16	16

*Median (minimum–maximum)

SUI, stress urinary incontinence; NA, not applicable; C_max_, maximum plasma concentration; t_max_, time to maximum plasma concentration; AUC_0–inf_, area under the plasma concentration versus time curve from time zero to infinity; t_1/2_, elimination half-time

## Discussion

The change in MUCP at 6 h after administering TAS-303 18 mg was slightly higher in the TAS-303 group than in the placebo group; however, the difference was not statistically significant.

Although there was no significant difference in the MUCP change for each urethral region between TAS-303 18 mg and placebo, the amount of change in the proximal urethra was larger with TAS-303 18 mg than with placebo. This result suggested that TAS-303 may have stronger pharmacological effects on the proximal urethra. There was just one AE not related to TAS-303; there were no safety concerns and the drug was well tolerated. The mean plasma concentration-time profile of TAS-303 in the present study was similar to that in a previous single-dose study in healthy adult men (unpublished data).

In a previous preclinical study in rats treated with duloxetine and TAS-303, TAS-303 caused a dose-dependent increase in basal urethral pressure in normal rats and in leak point pressure in rats with vaginal distention in vivo [10].

TAS-303 3 mg/kg produced an increase in urethral pressure in normal rats (by 38% compared with vehicle), whereas duloxetine 1 mg/kg increased the urethral pressure by 15% compared with vehicle. The increase in leak point pressure with TAS-303 3 mg/kg in vaginal distention (VD) rats was 26% and that with intravenous duloxetine 1 mg/kg was a 20% increase in VD rats. This result indicated that the effect of TAS-303 administration on urethral pressure was comparable to that of duloxetine. Therefore, a difference from placebo in the main end point (MUCP) in this study was expected.

Furthermore, the proximal urethra is mainly composed of urethral smooth muscle in which α1 receptors are concentrated [8, 9]. Therefore, we considered that TAS-303 administration could induce urethral contraction owing to α1 receptor stimulation by increasing the noradrenaline concentration in the urethra.

However, we did not find a statistically significant difference between TAS-303 and placebo in this study. The first reason for this result is that this study had a single-dose design, similar to a previous preclinical study [10] that showed a significant difference from placebo. The dose was selected as 18 mg based on the previous preclinical study [10] and a single-dose study in healthy adult men that evaluated safety (unpublished data). The mean plasma concentration-time profile of TAS-303 in the present study was similar to that in a previous single-dose study in healthy adult men and it reached the expected level. However, in a preclinical study, C_max_ was 17.7 ± 5 ng/mL at an effective dose of 2.7 mg/kg, whereas C_max_ for SUI female patients was 42.341 ng/ml, well above the preclinical concentration. Despite this, there was no significant difference from placebo. This may be related to differences in PK between animals and humans. The second reason is that the therapeutic range in humans may be higher than that in animals. In a 8–18 mg, repeated-dose study of TAS-303 conducted after this study, plasma concentration after once-daily repeated administration was maintained at five times the concentration reported after single-dose administration at the same doses [16]. Therefore, multiple doses may lead to sufficient blood concentration and have a significant difference from placebo. The third reason was that this study sample size may have been too small to permit confirmation of a significant difference. Fourth, as MUCP is more affected by the middle urethra than the proximal urethra, the measured pharmacological effect of TAS-303 on the proximal urethra may be lower than the actual effect [14]. Fifth, 11 out of 16 patient had menopause, and it may have negative impact on urethral contraction. Finally, in women with SUI, the urethral and para-urethral structures become progressively deficient. Intrinsic and extrinsic structural deficiencies result in a lower MUCP [14].

TAS-303 produced a larger change in the MUCP of the proximal urethra than the placebo, although the difference was not statistically significant. This suggested that TAS-303 may have a pharmacological effect. To determine a significant difference, the measurement of MUCP in the proximal urethra in a study with a steady-state blood concentration of TAS-303 and a larger sample size may be required.

In terms of safety, although TAS-303 has the mechanism of action of noradrenaline reuptake inhibitor, it rarely crosses the blood-brain barrier [10], which has little effect on the central nervous system. Therefore, unlike duloxetine and other noradrenaline reuptake inhibitor such as Atmoxetine [17], TAS-303 has little nausea related to the central nerve system and has shown a good safety profile.

The present study had some limitations. First, the sample size was small. Second, as this study included only Japanese women with SUI, the results cannot be generalized to other ethnic populations. Third, 11 out of 16 patient had menopause.

The findings of this study suggest that TAS-303 has pharmacological effects in the proximal urethra. A double-blind phase II study of TAS-303 18 mg and placebo in women with SUI is currently planned, and its results are awaited in the hope that this will contribute to the treatment of patients with SUI in the future.

## Electronic supplementary material

ESM 1 (DOCX 34 kb)

ESM 2 (DOCX 17 kb)

ESM 3 (DOCX 23 kb)
