# Supplementary material for: TAS-303 effects on urethral sphincter function in women with stress urinary incontinence: phase I study
Source: Int Urogynecol J. 2020 Oct 10;32(3):673–80. doi: 10.1007/s00192-020-04470-7 (PMC7902327; doi:10.1007/s00192-020-04470-7)
Supplement: Supplementary file 1 — (DOCX 34 kb) [file 192_2020_4470_MOESM1_ESM.docx]

**Supplementary Table 1.** Demographic and clinical characteristics

|  |  | **Group A**  **(N = 8)** | **Group B**  **(N = 8)** | **Total**  **(N = 16)** |
| --- | --- | --- | --- | --- |
| Age | Mean | 47.6 | 50.1 | 48.9 |
|  | Median [Min, Max] | 53.5 [23, 59] | 52.5 [32, 62] | 53.0 [23, 62] |
| Height | Mean (SD) | 158.54 (2.92) | 157.86 (4.60) | 158.20 (3.74) |
|  | Median [Min, Max] | 157.80  [155.4, 163.4] | 157.70  [149.1, 163.6] | 157.70  [149.1, 163.6] |
| Weight | Mean (SD) | 54.46 (7.76) | 55.01 (8.87) | 54.74 (8.06) |
|  | Median [Min, Max] | 52.80  [45.0, 70.4] | 53.85  [45.1, 73.3] | 53.25  [45.0, 73.3] |
| BMI [kg/m^2^] | Mean (SD) | 21.71 (3.42) | 22.05 (3.34) | 21.88 (3.27) |
|  | Median [Min, Max] | 20.80  [18.5, 28.9] | 20.68  [19.5, 29.7] | 20.69  [18.5, 29.7] |
| Type of urinary incontinence | SUI | 6 (75.0) | 4 (50.0) | 10 (62.5) |
|  | MUI | 2 (25.0) | 4 (50.0) | 6 (37.5) |
| Medical history | No | 8 (100.0) | 8 (100.0) | 16 (100.0) |
| Other symptoms | No | 8 (100.0) | 8 (100.0) | 16 (100.0) |

Analysis Set: FAS

BMI, body mass index; MUI, mixed urinary incontinence; SD, standard deviation; SUI, stress urinary incontinence
