# Supplementary material for: TAS-303 effects on urethral sphincter function in women with stress urinary incontinence: phase I study
Source: Int Urogynecol J. 2020 Oct 10;32(3):673–80. doi: 10.1007/s00192-020-04470-7 (PMC7902327; doi:10.1007/s00192-020-04470-7)
Supplement: Supplementary file 2 — (DOCX 17 kb) [file 192_2020_4470_MOESM2_ESM.docx]

**Supplementary Methods**

***Prohibited concomitant medications and therapies***

*From 4 weeks pre-dose to completion of follow-up:*

1) Drugs that affect urinary function

(a) Clenbuterol hydrochloride

(b) Drugs for disorders of urination (e.g., flavoxate hydrochloride, amino acid

combination drugs)

(c) Anticholinergics (e.g., propiverine hydrochloride, oxybutynin

hydrochloride, solifenacin succinate, tolterodine tartrate, fesoterodine

fumarate, imidafenacin, propantheline bromide, butropium bromide,

scopolamine butylbromide, kolantyl)

(d) β3-receptor agonists (mirabegron)

(e) α/β-receptor antagonists (e.g., labetalol hydrochloride, arotinolol

hydrochloride)

(f) α-receptor agonists (e.g., clonidine hydrochloride)

(g) Cholinergic agonists (e.g., bethanechol chloride, distigmine bromide)

(h) Antidepressants (e.g., imipramine hydrochloride, clomipramine

hydrochloride, amitriptyline hydrochloride, duloxetine hydrochloride,

milnacipran hydrochloride)

(i) Monoamine oxidase inhibitor (selegiline hydrochloride)

(j) Anxiolytics (e.g., diazepam, oxazolam, triazolam)

(k) Hypnotics (e.g., zopiclone, lormetazepam, flunitrazepam, estazolam)

(l) Sex hormones (e.g., chlormadinone acetate, oxendolone, gestonorone

caproate, allylestrenol)

(m) Other drugs expected to affect urinary function (including over the counter drugs) and the like (including supplements and health foods)

2) Physiotherapy (e.g., pelvic floor muscle exercises, feedback training, biofeedback

training, vaginal cone, electric stimulation therapy, magnetic stimulation therapy)

*From 7 days pre-dose to completion of follow-up:*

Foods and beverages containing grapefruit or St. John’s Wort

*From 3 days pre-dose to completion of follow-up*

Alcoholic beverages and caffeine-containing beverages

*From screening to completion of follow-up*

1) CYP3A4 substrate drugs with a narrow safety margin (e.g., alprazolam, trazodone,

amiodarone)

2) CYP3A4 inhibitors (e.g., itraconazole, voriconazole, clarithromycin)

3) Vaccinations (e.g., influenza vaccine)

***Randomization and blinding***

The study drug allocation manager prepared the treatment allocation and decoding documentation. Using Microsoft Office Excel, a unique random number corresponding to each patient ID code was generated. Of the random numbers generated, the eight largest numbers for the group assigned to “TAS-303 followed by placebo” and the remaining eight numbers for the group assigned to “placebo followed by TAS-303” were assigned to receive TAS-303 or placebo in a ratio of 1:1. The treatment allocation and decoding documentation were stored and managed by the study drug allocation manager, and the emergency codes were stored and managed by the investigator. The study drugs were placed into containers that were identical in appearance except that they were labeled with unique patient ID codes according to information derived from the randomization schedule, thereby blinding patients, raters at the study site, and the sponsor to study drug assignment, until code breaking.

***Urethral pressure measurement***

The urethral pressure profile (UPP) was measured by an 8-F dual microtip pressure transducer catheter (Unisensor AG, Attikon, Switzerland) with a pulling speed of 1 mm/s, and was recorded with a sampling rate of 250 Hz. The orientation of the pressure transducers was lateral at the three o’clock position. The simultaneous recording of both urethral and vesical pressures was performed to calculate urethral closure pressure and functional urethral length. Three successive profiles were measured with the patient resting and in the absence of bladder stimulation.
