# Supplementary material for: TAS-303 effects on urethral sphincter function in women with stress urinary incontinence: phase I study
Source: Int Urogynecol J. 2020 Oct 10;32(3):673–80. doi: 10.1007/s00192-020-04470-7 (PMC7902327; doi:10.1007/s00192-020-04470-7)
Supplement: Supplementary file 3 — (DOCX 23 kb) [file 192_2020_4470_MOESM3_ESM.docx]

**Supplementary Figure 1.** Study design

|  | Period 1 | Period 2 |
| --- | --- | --- |
| Group A | TAS-303 18 mg | TAS-303 placebo |
| Group B | TAS-303 placebo | TAS-303 18 mg |
